# Long-term effects of lumbar flexion versus extension exercises for chronic axial low back pain: a randomized controlled trial

**DOI:** 10.1038/s41598-024-51769-2

**Published:** 2024-02-01

**Authors:** Chul-Hyun Park, Jaewon Beom, Chun Kee Chung, Chi Heon Kim, Mi Yeon Lee, Myung Woo Park, Keewon Kim, Sun Gun Chung

**Affiliations:** 1 Department of Physical and Rehabilitation Medicine, Samsung Kangbuk Hospital, Sungkyunkwan University School of Medicine, Seoul, Republic of Korea; 2 Department of Rehabilitation Medicine, Seoul National University College of Medicine, Seoul National University Bundang Hospital, Seongnam, Republic of Korea; 3 Department of Physical Medicine and Rehabilitation, Chung-Ang University Hospital, Chung-Ang University College of Medicine, Seoul, Republic of Korea; 4 Department of Rehabilitation Medicine, Seoul National University College of Medicine, Seoul, Republic of Korea; 5 Department of Neurosurgery, Seoul National University Hospital and College of Medicine, Seoul, Republic of Korea; 6 Department of Neurosurgery, Seoul National University College of Medicine, Seoul, Republic of Korea; 7 Division of Biostatistics, Department of R&D Management, Samsung Kangbuk Hospital, Sungkyunkwan University School of Medicine, Seoul, Republic of Korea; 8 Department of Rehabilitation Medicine, Seoul National University College of Medicine, Seoul National University Hospital, 101 Daehak-Ro, Jongno-Gu, Seoul, 03080 Republic of Korea; 9 Institute of Aging, Seoul National University, Seoul, Republic of Korea

**Keywords:** Pain, Rheumatology

## Abstract

This study aimed to compare the long-term effects of flexion- and extension-based lumbar exercises on chronic axial low back pain (LBP). This was a 1-year follow-up of a prospective, assessor-blind, randomized controlled trial. Patients with axial LBP (intensity ≥ 5/10) for > 6 months allocated to the flexion or extension exercise group. Patients underwent four sessions of a supervised treatment program and were required to perform their assigned exercises daily at home. Clinical outcomes were obtained at baseline, 1, 3, 6 months, and 1-year. A total of 56 patients (age, 54.3 years) were included, with 27 and 29 in the flexion and extension groups, respectively. Baseline pain and functional scales were similar between both groups. The mean (± standard deviation) baseline average back pain was 6.00 ± 1.00 and 5.83 ± 1.20 in the flexion and extension groups, respectively. At 1-year, the average pain was 3.78 ± 1.40 and 2.26 ± 2.62 (mean between-group difference, 1.52; 95% confidence interval 0.56–2.47; *p* = *0.002*), favoring extension exercise. The extension group tended to have more improvements in current pain, least pain, and pain interference than the flexion group at 1-year. However, there was no group difference in worst pain and functional scales. In this controlled trial involving patients with chronic axial LBP, extension-based lumbar exercise was more effective in reducing pain than flexion-based exercises at 1-year, advocating lumbar extension movement pattern as a component for therapeutic exercise for chronic LBP.

Clinical Trial Registration No.: NCT02938689 (Registered on www.clinicaltrial.gov; first registration date was 19/10/2016).

## Introduction

Low back pain (LBP) is ranked as the primary cause of global disability^1^. Axial LBP, describing the pain confined to lower back region not traveling into the leg or feet^2,3^, is considered as one of the most common forms of chronic LBP, which may include non-specific LBP, discogenic back pain, facet joint syndrome, and so on^3^. There are various treatments for chronic axial LBP, such as lumbar exercises, anti-inflammatory medication, physical modalities, injection, and surgery^4,5^. Of these options, exercise therapy is one of mechanical strategies and the fundamental treatment for chronic axial LBP^4–9^. However, despite the numerous exercises prescribed for axial LBP, there remains no consensus regarding the most effective form of exercise. Therefore, an optimal exercise program for chronic axial LBP should be established. To formulate a safe and effective therapeutic exercise protocol, it is necessary to test each component of the protocol—type of exercise, movement pattern, intensity, frequency, duration and so on, using a well-designed, randomized controlled trial. In this study, the authors tested the direction of exercise as one of several aspects of movement pattern.

As the lumbar spine primarily moves in the sagittal plane, most low back exercises either flex or extend the lumbar spine, which can be categorized into flexion- or extension-based lumbar exercises^9–11^. These two exercises are widely utilized in clinical fields; however, they remain contradictory in practice and theory. Studies advocating flexion-based lumbar exercises indicated that lumbar lordosis was one of causes for LBP^11–15^. They suggested that patients should perform flexion exercises to eliminate or flatten lumbar lordosis, relieving nerve root compression by opening the intervertebral foramen and attenuating pressure on the posterior longitudinal ligaments and facet joints^14,16,17^. Contrarily, some studies advocated extension-based lumbar exercises to restore or maintain lumbar lordosis^18–20^, which is a unique structure developed to maintain an upright posture in humans^21^. Furthermore, they believed that lumbar lordosis is a prerequisite for human bipedal walking because it develops from the long-convex spine when a baby starts walking^22,23^. Though there has been a therapeutic approach advocating either flexion or extension exercise based on patient's preferred direction^24^, it is not widely endorsed because the exercise direction is determined only by patients’ symptom rather than anatomical structures responsible for individual symptoms^25^. With conflicting evidence and hypotheses on lumbar lordosis, the controversy over flexion- versus extension-based exercises for the treatment of chronic axial LBP remains unsolved^4,5,26^.

There are some previous studies comparing the effects of lumbar flexion versus extension exercises for back pain^27–32^. One previous study involving patients chronic mechanical LBP showed no group-difference in both flexion and extension modalities at 2-week follow-up^31^. Another study for chronic LBP patients compared the extension-based treatments such as hyperextension bracing and extension exercise and flexion-based treatments, which resulted in more pain improvements among extension treatment group at 1-month follow-up^27^. Furthermore, there are other past studies involving patients with acute/subacute LBP, which concluded that extension exercises showed better in disability and pain improvement at short-term follow-ups^28,29,32^. However, all these previous studies had too short follow-up duration after the flexion or extension exercise intervention. Most of studies did not randomly allocate the study participants. To the best of our knowledge, there is no long-term follow-up, prospective, randomized controlled trial regarding exercise directions focused only on chronic axial LBP.

Therefore, this study aimed to compare the effects of lumbar flexion- and extension-based exercises on chronic axial LBP at 1-year follow-up.

## Results

### Baseline characteristics

**Figure 1:**
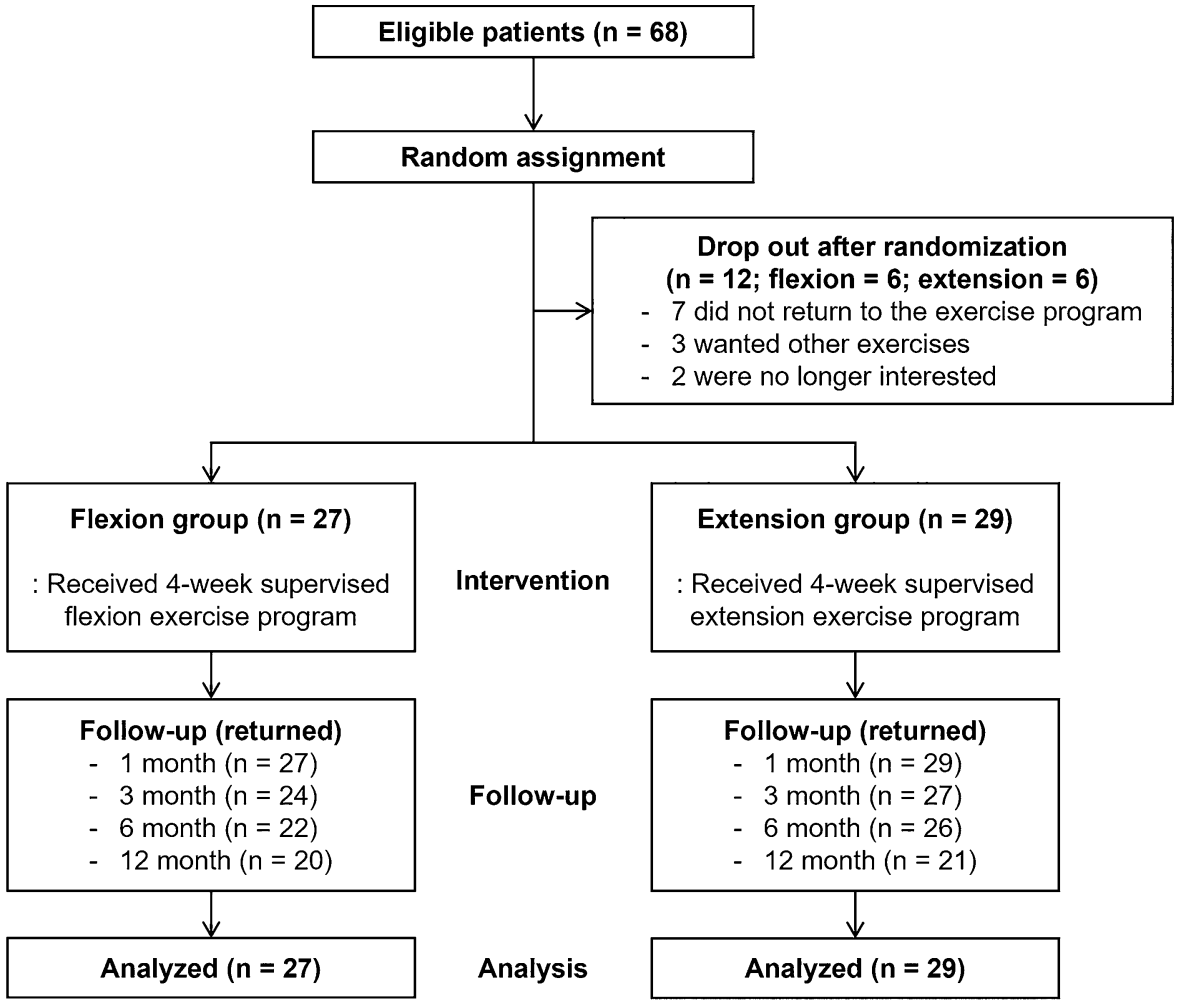
Flow diagram of the study.

Sixty-eight participants satisfying all the criteria were randomly assigned to two groups. Each group had six dropouts after randomization, leaving 27 and 29 patients in the flexion and extension groups, respectively, who underwent four sessions of the supervised exercise program (Fig. 1). The patients’ mean (± SD) age was 54.32 ± 14.41 years, 30.36% were men, and the mean BMI was 23.34 ± 2.63. The mean average pain of the lower back region was 6.00 ± 1.00 in the flexion group and 5.83 ± 1.20 in the extension group (*p* = *0.563*). The mean duration of axial LBP were 13.6 ± 4.6 in flexion group, 13.0 ± 4.4 in extension group, respectively (*p* = *0.622*). The baseline demographic and clinical characteristics including pain scores and functional scales were similar between the two groups (Table 1).

**Table 1:** Baseline characteristics.

Variables	Total (n = 56)	Flexion group (n = 27)	Extension group (n = 29)
Age (years)	54.32 ± 14.41	55.44 ± 13.18	53.28 ± 15.63
Sex (male, %)	17 (30.36)	8 (29.63)	9 (31.03)
Height (cm)	161.81 ± 8.01	161.04 ± 7.2	162.55 ± 8.79
Weight (kg)	61.21 ± 9.08	59.64 ± 8.67	62.73 ± 9.37
BMI (kg/m^2^)	23.34 ± 2.63	22.98 ± 2.75	23.70 ± 2.51
History of spinal injection	8 (14.29)	4 (14.81)	4 (13.79)
Duration of axial LBP (month)	13.3 (4.4)	13.6 (4.6)	13.0 (4.4)
Underlying diseases			
Diabetes mellitus (%)	3 (5.4)	1 (3.7)	2 (6.9)
Hypertension (%)	11 (19.6)	5 (18.5)	6 (20.7)
Hyperlipidemia (%)	7 (12.5)	5 (18.5)	2 (6.9)
Heart disease (%)	2 (3.6)	0 (0)	2 (6.9)
Kidney disease (%)	1 (1.8)	1 (3.7)	0 (0)
Baseline score of outcome measure			
Primary outcome			
Average pain*	5.91 ± 1.10	6.00 ± 1.00	5.83 ± 1.20
Secondary outcome: pain subscales			
Current pain*	4.95 ± 1.53	4.78 ± 1.78	5.10 ± 1.26
Worst pain*	6.46 ± 1.36	6.74 ± 1.20	6.21 ± 1.47
Least pain*	2.48 ± 1.73	2.48 ± 1.25	2.48 ± 2.10
Pain interference^†^	4.41 ± 2.0	4.94 ± 2.05	3.93 ± 1.86
Functional scales			
ODI^‡^	18.39 ± 9.89	20.19 ± 11.07	16.7 ± 8.50
EQ-5D^§^	0.73 ± 0.12	0.70 ± 0.13	0.75 ± 0.10
PASE^¶^	103.03 ± 55.04	108.5 ± 66.75	98.14 ± 42.66

Values are means ± standard deviation or no. (%) of participants in each group. There were no significant differences between the groups.

*BMI* body mass index, *BPI* brief pain inventory, *EQ-5D* EuroQol-5D, *ODI* Oswestry disability index, *PASE* physical activity scale for the elderly.

*Scores on the pain scales of BPI range from 0 to 10, with higher scores indicating more pain.

^†^Scores on the pain interference scale based on BPI range from 0 to 10, with higher scores indicating greater interference in daily functioning due to pain.

^‡^ODI range from 0 to 100, with higher scores representing greater disability associated with low back pain.

^§^EQ-5D range from − 0.594 to 1, with higher scores indicating higher health utility.

^¶^PASE range from 0 to 793, with higher scores indicating greater physical activity.

### Primary outcome

**Figure 2:**
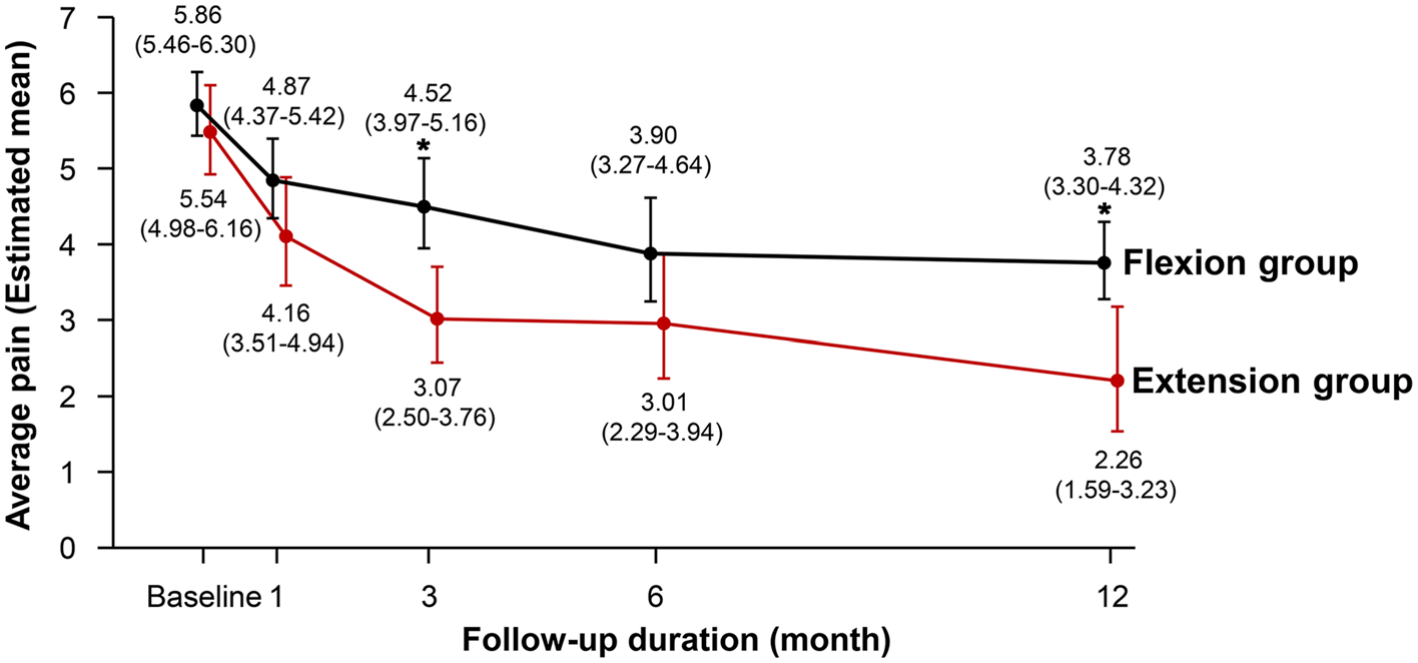
Average back pain score over the 12-month follow-up period. The average back pain scores ranged from 0 to 10, with higher scores indicating more severe pain. The values in parentheses are 95% confidence intervals. All the patients in the flexion (n = 27) and extension (n = 29) groups were included in the analysis. Asterisk indicates the significant difference between the two groups at each time point.

The average (± SD) pain scores at 1 year were 3.78 ± 1.40 in the flexion group and 2.26 ± 2.62 in the extension group (mean between-group difference, 1.52; 95% CI 0.56–2.47; *P* = 0.002), favoring extension exercise (Table 2; Fig. 2). A significant difference in the average pain score persisted after adjusting for age, sex, and BMI (adjusted* P* = 0.004). These results remained unchanged in the pre-specified sensitivity analyses performed with imputation for missing values using the mean of non-missing items (Supplementary Table 1). The average pain scores at all time points are listed in Supplementary Tables 2 and 3.

**Table 2:** Primary and secondary pain outcomes at 1 year.

	Flexion group (n = 27)	Extension group (n = 29)	Unadjusted mean between group difference (95% CI)	Adjusted mean between group difference (95% CI)*
Primary outcome				
Average pain	3.78 (3.30–4.32)	2.26 (1.59–3.23)	1.52 (0.56–2.47)^†^	1.38 (0.43–2.32)^†^
Secondary outcome: pain subscales				
Current pain	3.64 (3.13–4.25)	1.83 (1.20–2.79)	1.81 (0.86–2.77)^†^	1.73 (0.76–2.70)^†^
Worst pain	4.58 (3.96–5.30)	4.06 (3.25–5.07)	0.52 (− 0.60 to 1.65)	0.51 (− 0.63 to 1.64)
Least pain	1.82 (1.47–2.24)	0.80 (0.49–1.31)	1.01 (0.46–1.56)^†^	0.92 (0.35–1.48)^†^
Pain interference	3.36 (2.77–4.06)	2.17 (1.48–3.16)	1.19 (0.15–2.23)^†^	1.20 (0.16–2.24)^†^

Generalized linear mixed models comparing between-group difference were used for multiple comparisons. Values were presented as least-squares mean (95% CI).

Scores of primary and secondary outcome range from 0 to 10, with higher values indicating more severe pain or interference on daily life activity.

*CI* confidence interval.

*Adjusted mean between group differences at the time point were estimated after adjustments for age, sex, and body mass index at baseline.

^†^*P* for between-group difference < 0.05.

### Secondary outcome

**Figure 3:**
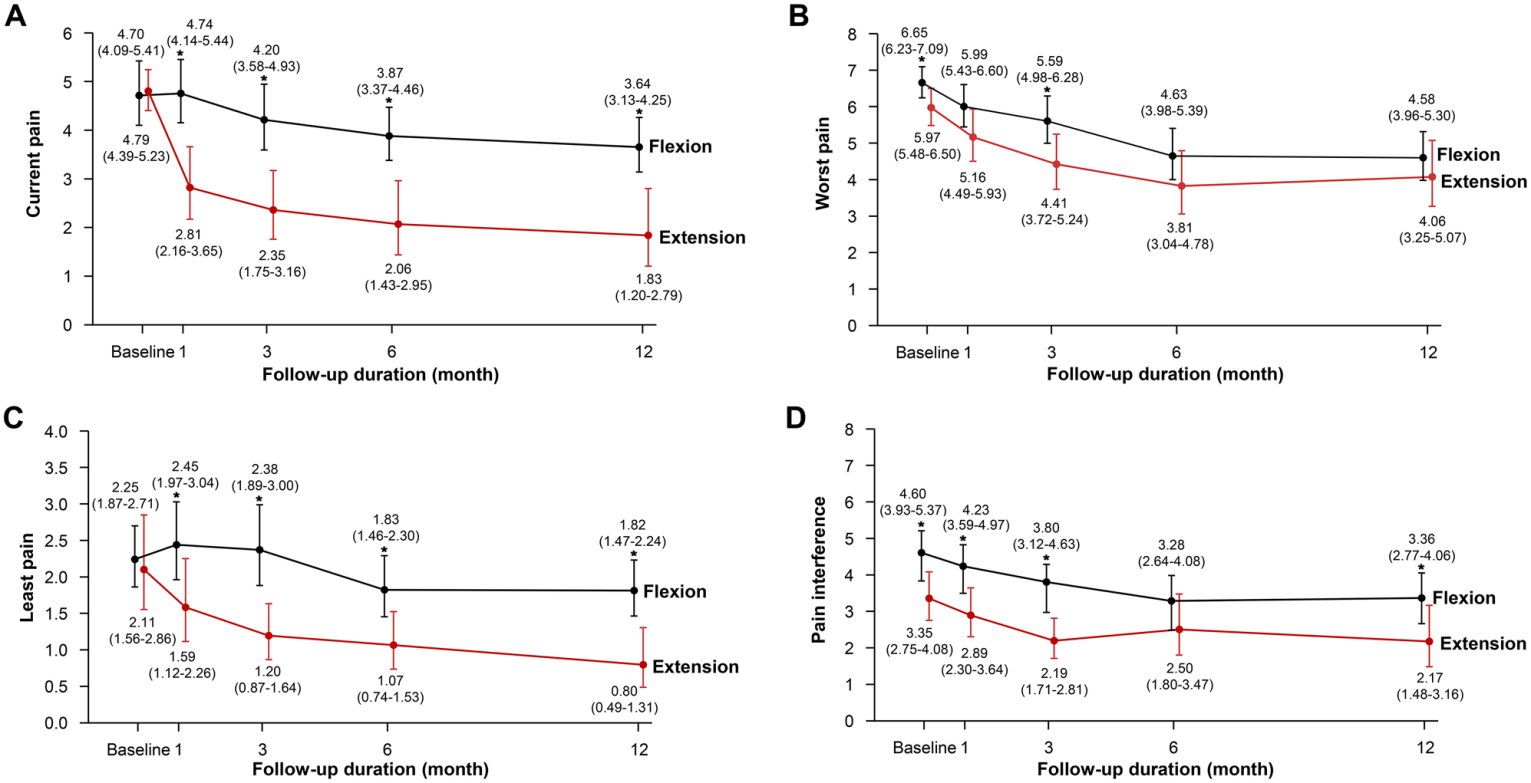
Secondary outcome on (**A**) current pain, (**B**) worst pain, (**C**) least pain, and (**D**) pain interference from brief pain inventory over the 12-month follow-up period. The scores ranged from 0 to 10, with higher scores indicating more severe pain and symptoms. The values in parentheses are 95% confidence intervals. Asterisk indicates the significant difference between the two groups at each time point.

The mean between-group differences at 1 year for current pain was 1.81 (95% CI 0.86–2.77), 1.01 for least pain (95% CI 0.46–1.56), and 1.19 for pain interference (95% CI 0.15–2.23); patients in the extension group showed greater improvement in the pain subscales than patients in the flexion group (Table 2; Fig. 3). The results were consistent after adjusting for age, sex, and BMI. There were no significant between-group difference for worst pain. Furthermore, functional scales such as ODI, EQ-5D, and PASE were similar between the flexion and extension group at 1 year (Table 3; Supplementary Tables 4 and 5).

**Table 3:** Functional scales at 1 year.

	Flexion group (n = 27)	Extension group (n = 29)	Unadjusted mean between group difference (95% CI)	Adjusted mean between group difference (95% CI)*
ODI	13.16 (10.54–16.44)	10.67 (7.66–14.85)	2.49 (− 2.09 to 7.08)	2.41 (− 2.10 to 6.93)
EQ-5D	0.78 (0.75–0.81)	0.80 (0.74–0.86)	− 0.02 (− 0.09 to 0.05)	− 0.02 (− 0.08 to 0.04)
PASE	118.18 (94.90–147.17)	131.40 (100.90–171.12)	− 13.22 (− 56.55 to 30.10)	− 13.93 (− 55.94 to 28.07)

Generalized linear mixed models comparing between-group difference were used for multiple comparisons. Values were presented as least-squares mean (95% CI).

*CI* confidence interval, *EQ-5D* EuroQol life-quality index, *ODI* Oswestry disability index, *PASE* physical activity scale for the elderly.

*Adjusted mean between group differences at the time point were estimated after adjustments for age, sex, and body mass index at baseline.

There were no serious adverse events during the 12-month follow-up period in either group (Table 4). The incidence of non-serious adverse events related to their exercise programs was similar between both groups. All adverse events were mild and transient, without the need for additional treatment. The most frequently reported adverse event was LBP, reported by six (22.2%) and seven (24.1%) patients in the flexion and extension groups, respectively. The participants reported a slight initial increase in lower back pain relieved by the second or third week of their allocated exercise program. Transient sciatica or lower limb radiating pain was reported in three (11.1%) patients only in the flexion group. The between-group adherence rate was similar throughout the study period (Supplementary Table 6).

**Table 4:** Adverse events over the 1-year follow-up period in the flexion and extension groups.

	Flexion group (n = 27)	Extension group (n = 29)	*P* value
Deaths	0	0	
Serious adverse effects	0	0	
Non-serious adverse effects			
Low back pain	6 (22.2)	7 (24.1)	0.808
Sciatica or lower limb radiating pain	3 (11.1)	0	0.065
Posterior neck pain	1 (3.7)	1 (3.4)	0.959
Pain in wrist, elbow, or shoulder joint	1 (3.7)	3 (10.3)	0.335
Pain in hip, knee, or ankle joint	2 (7.4)	0	0.136
Unrelated fall or other trauma	0	1 (3.4)*	0.330

Values represent number (%) of participants in each group. There were zero withdrawals from the trial related to any form of adverse effects.

*Ankle injury by fall down.

## Discussion

We observed that lumbar extension exercise was more effective than flexion exercise in improving outcomes at 1 year, as assessed by the average back pain score. The extension group showed a higher improvement in pain subscales, such as least pain, current pain, and pain interference, than those in the flexion group but not in the functional scale as compared with the flexion group. No between-group differences were found in the functional scale and adverse events. However, radiating pain in the lower limb occurred more often in the flexion group. To the best of our knowledge, this is the first report to demonstrate that lumbar extension exercise to augment lumbar lordotic curvature is better than flexion-based lumbar exercise to diminish the curvature in reducing chronic axial LBP in a 1-year follow up, randomized controlled trial.

### Efficacy of lumbar extension exercises on pain reduction

Although many previous studies were conducted for the effects of exercise on LBP, there are only a several studies which directly compared the effect of lumbar flexion and extension exercise for chronic LBP^28–30^. Elnaggar et al. conducted the 1-month trial comparing flexion versus extension exercise among 56 patients (aged from 20 to 50 years) with chronic LBP for 3 months or more^31^. The result showed that both exercise modalities provided significant improvements in back pain severity, but there was no significant difference between the groups. This previous result is in line with our study, both studies presented that the flexion and extension group showed the improvements throughout the period. Furthermore, in the present study, at 1 month follow-up, the average back pain was similar between groups (Fig. 2). However, the major difference in our study is that further follow ups were performed after 3, 6 months and 1 year in an assessor-blinded, prospective, randomized controlled design, which resulted in more improvements in pain reduction among extension group than flexion group at 1 year.

There are a few explanations to this. First, lumbar extension exercise is an efficient way to maintain the lordotic curvature of lumbar spine. Contrarily, flexion exercise aims to eliminate the lordotic curve. Lumbar lordotic curvature is a key component of sagittal alignment^33^. A decrease in the lordotic curvature of lumbar spine is closely related to anterior sagittal imbalance, affecting the intervertebral discs with prolonged stress and loading^34^. This stress concentration of intervertebral discs with decreased sagittal alignment can contribute to a degenerative cascade of disc diseases^35^. A previous meta-analysis have reported that patients with LBP had decreased lumbar lordosis than healthy participants^36^. Takeda et al.^37^ demonstrated that the loss of lumbar lordotic curvature occurred with aging in a 10-year longitudinal study. Therefore, restoring lumbar lordotic curvature, a major determinant of sagittal balance, appears to be favorable in managing chronic LBP patients.

Second, repetitive flexion movement of lumbar spine can lead to posterior displacement of the nucleus pulposus (NP) with thinning of posterior annulus fibrosus, entailing a herniated disc disorder combined with an annulus tear. Previous studies proved that a lumbar flexion movement of an intervertebral disc induced posterior migration of the NP and decreased anterior disc height compared with an extension movement using magnetic resonance imaging in both living participants and cadaveric specimens^38–40^. Nazari et al.^19^ reported that decreasing the anterior disc’s height causes the posterior disc segment to stretch in a flexed posture, which can cause the NP to become closer to the spinal canal and bulge outwards, causing pain related to herniated disc disorder.

Lastly, the intradiscal pressure on the posterior annulus can be increased in flexion-based exercises. Interestingly, in 1976, Nachemson et al. investigated the effect of active flexion exercises and passive flexed posture on intra-discal pressure, which significantly increased in both conditions^41^. For instance, the intra-discal pressure of sit-up exercise, which is a part of our flexion-based exercise program, appeared 2.1 times higher than that of standing position^41^. Adams et al. demonstrated by a cadaveric experiment that compressive stress and loading on intervertebral discs could induce progressive structural changes of intervertebral disc and endplates with protrusions of the NP^42^. Furthermore, a bulk of extrusion of the NP occurred when a lumbar spine was heavily loaded at a flexion angle. Therefore, extension-based lumbar exercises can be effective in treatment of chronic LBP patients.

### Exercise adherence and natural course of healing on LBP improvement

The difference in average back pain between the flexion and extension groups was the highest at the 3-month visit, a period of high adherence to lumbar exercises. This 3-month high-compliance period showed more improvements in average back pain in the extension group than in the flexion group. However, after a 3-month low-adherence period, indicating a poor effect of lumbar exercises, the average pain score was influenced by the intervertebral disc’s natural healing process, which seems considerable. Thus, to maximize the treatment effect of lumbar exercise for LBP, patient adherence is presumed to be clinically important. Therefore, as previous studies have recommended supervised exercises for chronic LBP ^43,44^, we strongly suggest that lumbar extension exercises combined with great supervision should be advocated for patients with chronic axial LBP.

In this trial, the two exercise groups’ adherence was excellent in the first 3 months. However, the adherence rates decreased to approximately 60% from 4 to 6 months of follow-up (Supplementary Table 6). Nevertheless, the average back pain decreased in both groups after 12 months. A possible reason for this is mechanical LBP's self-recovery potential. The common etiology of LBP is intervertebral disc disorders, such as annular tears or herniated discs. It has been demonstrated that axial LBP is closely related to outer annular tear by intra-operative tissue stimulation ^45,46^, or by relating cadaveric findings with back pain in life ^47^. Therefore, healing the torn outer annulus should be the primary focus on managing axial LBP.

### Safety concerns regarding lumbar exercises

A few adverse events were reported in this study. However, all adverse events were mild and transient, suggesting that flexion and extension-based lumbar exercises are well-tolerated interventions for chronic axial LBP. Although the between-group incidence of adverse events was comparable, transient sciatica occurred only in the flexion group (11.1%, 3/27 patients), which deserves further attention.

The exact pathomechanism of sciatica’s evolution in the flexion group is unknown. However, there are possible explanations for the presence of sciatica in the flexion group. First, repetitive flexion movements can induce irritation of nerve roots, leading to sciatica. Schnebel et al. demonstrated that the compressive force and tension of nerve roots were increased by flexion of lumbar spine at the lower lumbar level but were decreased by an extension of lumbar spine ^48^. This sciatica induced by lumbar flexion can also be explained using a straight leg raise test, which provokes irritation of lower lumbar roots when raising the leg^49^. Therefore, prescribing lumbar exercises of flexion movement should be avoided in patients with LBP, especially those with a history of radiating symptoms. Moreover, further research should investigate the potentially harmful effects of lumbar flexion exercises.

### Limitations

This study has several limitations. First, the number of participants was small. Participants were recruited based on strict inclusion and exclusion criteria from tertiary-care hospitals to minimize individual variation among patients. Second, the first 4-week of education and supervised exercises may have been insufficient to maximize exercise adherence. As the proportion of adherent patients decreased during the follow-up period, expanding the duration of supervised exercises would maximize the treatment effects of lumbar exercises. Third, the etiology of chronic LBP was not evaluated by MR (magnetic resonance) imaging to confirm the patho-anatomical cause of the pain. The participants were only included as the criteria by clinical symptoms presented with axial LBP pain. Therefore, participants could have heterogenous causes for LBP, which needs to be cautious in interpreting the result of our data. Fourth, the measurement of degree of lordotic curve was not measured which could be important outcome in this study. In a future study, the changes of lordotic angle should be investigated after spine flexion or extension exercise. Lastly, the prescription of our exercise intervention was not individualized to each patient. In the clinical setting, to maximize the improvement of pain and disability, an individualized exercise prescription should be considered based on the thorough physical examination and lumbar MRI or computed tomography.

## Conclusions

Extension-based lumbar exercises improved chronic axial LBP more than lumbar flexion exercises at 1-year follow-up. Radiating pain in the lower limbs occurred only in the flexion group. The result of this clinical trial implicates that lumbar extension movement pattern to restore lumbar lordotic curvature should be included in developing pertinent exercise therapy for chronic axial LBP.

## Methods

### Study participants

This study was a 1-year follow-up of a two-center, prospective, assessor-blind, randomized controlled trial that compared the effectiveness of flexion- versus extension-based lumbar exercises in chronic axial LBP. Patients with chronic axial LBP were recruited from two large tertiary care university hospitals through local advertisements. The inclusion criteria were (1) a history of chronic axial LBP, defined as pain confined to lumbar region where above the gluteal folds and below the costal margin ^50,51^; (2) duration of symptoms persisting for ≥ 6 months^52^, which were poorly responsive to conservative treatments (e.g., physical modalities, anti-inflammatory medication, epidural, or facet joint steroid injections); and (3) average pain intensity by an 11-point numeric rating scale (NRS; rating from 0 to 10) of ≥ 5 over the last 2 weeks as previously defined^53^.

The exclusion criteria were (1) lumbar spine surgery, including discectomy, laminectomy, or fusion operation; (2) spondylolisthesis or retrolisthesis on whole spine radiography; (3) any spine intervention such as an epidural, facet joint steroid injection, or neuroplasty within 3 months; (4) predominant sciatica or radiating leg pain more than LBP; (5) neurologic or inflammatory disorder; or (6) poor cooperation for performing lumbar exercises due to any medical conditions (e.g., cardiorespiratory illness or severe psychiatric disorders).

### Randomization and blinding

An initial screening interview for demographic information and diagnostic whole-spine radiography, including anteroposterior and lateral views, was performed before randomization. Eligible patients were randomly assigned to either the flexion- or extension-based exercise groups in a 1:1 ratio using a block randomization method from the website of the Medical Research Collaborating Center (MRCC) of Seoul National University Hospital, which was not involved in the trial.

Participants were firstly screened by research assistants with > 3 years of experience and they were screened again by musculoskeletal specialized physiatrists (with PhD degree) with at least 10 years of experience (C.-H.P. and J.B.). The research assistants performed outcome assessments and were blinded to the trial-group assignments. Although blinding of physiotherapists and patients was not possible due to the nature of exercise program, the blinded assistants evaluated each patient using a structured questionnaire addressing any issues that might imply trial allocation.

### Intervention

The participants received four individual face-to-face sessions of a 30-min supervised treatment program (flexion- or extension-based exercise) once a week, with musculoskeletal-specialized physiotherapists as at least > 10 years of experience. These sessions included instructions and principles for the allocated exercises and behavioral components to encourage adherence. At the initial session, patients were shown images and given protocols for the assigned exercises. Patients were then instructed to start their daily home exercises for at least 30 min/day and were asked to continue them until the end of the year-long study period. Patients attended up to four individual sessions over the initial 4-week period. The patients could undergo an additional one to two sessions at the time of the 3- and 6-month reassessment period if further instruction was required.

The patients allocated to the flexion or extension group received the theoretical information regarding the direction of the spine and performed their specific exercise according to the direction of allocated exercises. The flexion-based lumbar exercises comprised a set of exercises focusing on enhancing lumbar flexion to minimize lumbar lordosis (Supplementary Fig. 1)^11,29,31^. Extension-based lumbar exercises were emphasized with lumbar extension movements as opposed to flexion exercises (Supplementary Fig. 2)^10,29,31^. The flexion exercises comprised of pelvic tilt, knee-to-chest, trunk flexion, and forward bending with hip flexor stretch exercises. The extension exercises were prone lying flat, prone propped on elbows, prone propped on hands, and standing lumbar extension exercises. Each group were required to perform all four positions of allocated exercise program in one session starting from the first week of exercise intervention. Each session took at least 30 min. Patients were to perform their daily home exercises for at least 30 min per day until the end of the year-long study period.

### Assessments and outcomes

Clinical outcomes were assessed at baseline, the end of the supervised exercise session (1 month), and 3 months, 6 months, and 1 year after randomization. The patients visited the clinic at the time of reassessment. The primary outcome was the average pain score of lower back region at 1 year, which measured the average intensity of pain during the past 24 h on a scale from 0 to 10.

The secondary outcomes included pain subscales from the Brief Pain Inventory (BPI). The BPI is a widely utilized self-administered questionnaire and validated NRS for assessment of pain intensity by handing the structured questionnaire or by asking each question verbally^54–56^. Scores on the pain scales of BPI range from 0 to 10, with higher scores indicating more pain. Patients were asked to response to the questionnaire for the intensity of each types of pain such as average pain, current pain, worst pain, and least pain based on the past 24 h at the time of clinic assessment by the blinded research assistant. Pain severity items such as (1) current pain, (2) worst pain, and (3) least pain in the lumbar region were included as secondary outcome, which evaluated specific pain intensity on a scale of 0–10. Pain interference was evaluated based on seven categories of pain interference in daily life activities^54^. Functional scales such as the Oswestry disability index (ODI), EuroQol life-quality index (EQ-5D), and Physical Activity Scale for the Elderly (PASE) were evaluated^57,58^.

Adherence to the exercise protocol was monitored at each assessment visit (1, 3, and 6 months and 1 year) and by phone calls at 2, 4, and 5 months. Patients were asked if they performed their assigned exercises and were asked regarding the frequency and duration of exercises per week. Adherent participants were defined as those who completed their exercises over 30 min at least four times a week. The occurrence of adverse events was assessed by asking whether the participant had significant pain aggravation during exercise.

### Sample size estimation

The sample size was originally calculated for the primary outcome variable, the average back pain score using NRS, using the Power Analysis and Sample Size software (http://www.ncss.com). We considered the values from a previous exercise intervention randomized controlled trial with similar population and protocols^59^, which reported that the mean change in back pain was 3.35 and 1.63 and the standard deviations (SDs) were 2.39 and 2.06 in the intervention and control groups, respectively. Based on these findings, considering 2.2 as an acceptable SD, the power was set at 0.80, type I error α was 0.05, and type II error β was 0.20 with a two-sided significance level of 0.05^60^. Assuming a dropout percentage of 20%, the calculated sample size was 34 for each group, and the total size was 68 participants.

### IRB approval and clinical trial registration

The study protocol and ethics approval were obtained from the Institutional Review Board of Seoul National University Hospital (no. H-1607-199-782). This trial was registered in the Clinical Trials Registry (ClinicalTrials.gov; no. NCT02938689) before recruitment. First registration date was 19/10/2016. All methods were performed in accordance with the relevant guidelines and regulations. Before study commencement, participants were informed of the clinical trial, provided written informed consent, and coordinators determined if the participants met the eligibility criteria.

### Statistical analysis

Comparisons of the baseline characteristics between the groups were performed using an independent t-test or chi-square test. All analyses were performed on patients receiving a 4-week supervised exercise treatment program and were based on the intention-to-treat principle. To analyze the primary and secondary outcomes, a generalized linear mixed model was used to compare the two groups, with adjustment for multiple comparisons. Results were presented as the least-squares mean and 95% confidence interval (CI), including the mean differences between groups at time points controlling for age, sex, and body mass index (BMI) at baseline. We used the last observation carried forward (LOCF) approach for the imputation of missing data. In addition, we conducted a sensitivity analysis using the mean imputation method instead of the LOCF to assess between-group differences in primary and secondary outcomes^61^. IBM SPSS version 27 (IBM Corp., Armonk, NY, USA) and STATA version 17.0 (StataCorp LP, College Station, TX, USA) were used for all analyses. Statistical significance was defined as a two-tailed *P* value < 0.05.

### Supplementary Information

Supplementary Information.

## Data Availability

The datasets of the current study cannot be made openly available to protect the medical information of participants. However, the corresponding author can provide the dataset on a reasonable request.
