# Supplementary material for: Long-term effects of lumbar flexion versus extension exercises for chronic axial low back pain: a randomized controlled trial
Source: Sci Rep. 2024 Feb 1;14:2714. doi: 10.1038/s41598-024-51769-2 (PMC10834418; doi:10.1038/s41598-024-51769-2)
Supplement: Supplementary file 1 — Supplementary Information. [file 41598_2024_51769_MOESM1_ESM.pdf]

Supplementary Digital Materials

Supplementary Table 1. Primary and secondary pain outcomes at 1-year assessed by mean imputation method

Supplementary Table 2. Primary and secondary pain outcomes at all measure points assessed by LOCF method

Supplementary Table 3. Primary and secondary pain outcomes at all measure points assessed by mean imputation method

Supplementary Table 4. Functional scales at all measure points assessed by LOCF method

Supplementary Table 5. Functional scales at all measure points assessed by mean imputation method

Supplementary Table 6. Adherence rate over 1-year follow up

Supplementary Fig 1. Exercise interventions based on the lumbar flexion.

Supplementary Fig 2. Exercise interventions based on the lumbar extension.

Supplementary Table 1. Primary and secondary pain outcomes at 1-year assessed by mean imputation method

	Flexion group	Extension group	Unadjusted mean between-group difference (95% CI)	Adjusted mean between-group difference (95% CI)*
**Primary outcome**				
Average pain	3.65 (3.16–4.22)	1.96 (1.46–2.62)	1.7 (0.92–2.48)†	1.65 (0.85–2.44)†
**Secondary outcome: pain subscales**				
Current pain	3.69 (3.11–4.38)	1.61 (0.95–2.71)	2.08 (1.04–3.13)†	2.03 (0.96–3.09)†
Worst pain	4.4 (3.67–5.29)	3.95 (3.08–5.07)	0.45 (-0.82–1.72)	0.4 (-0.92–1.72)
Least pain	1.72 (1.37–2.18)	0.75 (0.41–1.37)	0.97 (0.37–1.58)†	0.89 (0.27–1.50)†
Pain interference	3.39 (2.72–4.22)	1.74 (1.12–2.72)	1.64 (0.57–2.71)†	1.66 (0.55–2.78)†

Generalized linear mixed models comparing between-group differences were used for multiple comparison. Values were presented as least-squares mean (95% CI).

Scores of primary and secondary outcome range from 0–10, with higher values indicating more severe pain or interference on daily activity.

CI: confidence interval.

*Adjusted mean between-group difference at the time point was estimated after adjustments for age, sex, and body mass index at baseline.

†p for between-group difference <0.05.

Supplementary Table 2. Primary and secondary pain outcomes at all measure points assessed by LOCF method

	Unadjusted mean (95% CI)		Flexion group versus Extension group	
	Flexion group	Extension group	Unadjusted mean differences (95% CI)	Adjusted mean differences (95% CI)*
**Primary outcome**				
**Average pain**				
Baseline	5.86 (5.46–6.30)	5.54 (4.98– 6.16)	0.33 (-0.40 – 1.05)	0.57 (-0.25 – 1.39)
1 month	4.87 (4.37–5.42)	4.16 (3.51–4.94)	0.7 (-0.18 – 1.59)	0.59 (-0.25 – 1.43)
3 months	4.52 (3.97–5.16)	3.07 (2.50–3.76)	1.46 (0.60 – 2.32)†	1.39 (0.56 – 2.23)†
6 months	3.90 (3.27–4.64)	3.01 (2.29–3.94)	0.89 (-0.17 – 1.95)	0.76 (-0.31 – 1.82)
12 months	3.78 (3.30–4.32)	2.26 (1.59–3.23)	1.52 (0.56 – 2.47)†	1.38 (0.43 – 2.32)†
**Secondary outcome: pain subscales**				
**Current pain**				

Baseline	4.70 (4.09–5.41)	4.79 (4.39–5.23)	-0.09 (-0.87–0.69)	-0.14 (-0.89–0.61)
1 month	4.74 (4.14–5.44)	2.81 (2.16–3.65)	1.93 (0.95–2.91)†	1.82 (0.87–2.77)†
3 months	4.20 (3.58–4.93)	2.35 (1.75–3.16)	1.85 (0.88–2.81)†	1.75 (0.78–2.71)†
6 months	3.87 (3.37–4.46)	2.06 (1.43–2.95)	1.82 (0.90–2.74)†	1.73 (0.81–2.66)†
12 months	3.64 (3.13–4.25)	1.83 (1.20–2.79)	1.81 (0.86–2.77)†	1.73 (0.76–2.7)†
**Worst pain**				
Baseline	6.65 (6.23–7.09)	5.97 (5.48–6.50)	0.68 (0.02–1.34)†	0.78 (0.06–1.50)†
1 month	5.99 (5.43–6.60)	5.16 (4.49–5.93)	0.83 (-0.10–1.75)	0.89 (-0.04–1.81)
3 months	5.59 (4.98–6.28)	4.41 (3.72–5.24)	1.18 (0.18–2.18)†	1.17 (0.18–2.15)†
6 months	4.63 (3.98–5.39)	3.81 (3.04–4.78)	0.82 (-0.29–1.93)	0.82 (-0.27–1.92)
12 months	4.58 (3.96–5.30)	4.06 (3.25–5.07)	0.52 (-0.60–1.65)	0.51 (-0.63–1.64)
**Least pain**				
Baseline	2.25 (1.87–2.71)	2.11 (1.56–2.86)	0.14 (-0.62–0.91)	0.04 (-0.70–0.77)

1 month	2.45 (1.97–3.04)	1.59 (1.12–2.26)	0.85 (0.08–1.63)†	0.72 (-0.03–1.47)
3 months	2.38 (1.89–3.00)	1.20 (0.87–1.64)	1.19 (0.52–1.85)†	1.06 (0.42–1.71)†
6 months	1.83 (1.46–2.30)	1.07 (0.74–1.53)	0.76 (0.20–1.33)†	0.66 (0.11–1.22)†
12 months	1.82 (1.47–2.24)	0.80 (0.49–1.31)	1.01 (0.46–1.56)†	0.92 (0.35–1.48)†
**Pain interference**				
Baseline	4.60 (3.93–5.37)	3.35 (2.75–4.08)	1.24 (0.27–2.22)†	1.30 (0.29–2.32)†
1 month	4.23 (3.59–4.97)	2.89 (2.30–3.64)	1.33 (0.38–2.29)†	1.35 (0.37–2.33)†
3 months	3.80 (3.12–4.63)	2.19 (1.71–2.81)	1.61 (0.69–2.53)†	1.61 (0.67–2.56)†
6 months	3.28 (2.64–4.08)	2.50 (1.80–3.47)	0.78 (-0.31–1.87)	0.78 (-0.30–1.87)
12 months	3.36 (2.77–4.06)	2.17 (1.48–3.16)	1.19 (0.15–2.23)†	1.20 (0.16–2.24)†

Generalized linear mixed models comparing between-group difference were used for multiple comparison. Values were presented as least-squares mean (95% CI).

Scores of primary and secondary outcome range from 0 to 10, with higher values indicating more severe pain or interference on daily activity.

LOCF: last observation carried forward; CI: confidence interval.

*Adjusted mean between-group difference at the time point was estimated after adjustments for age, sex, and body mass index at baseline.

†p for between-group difference <0.05.

Supplementary Table 3. Primary and secondary pain outcomes at all measure points assessed by mean imputation method

	Unadjusted mean (95% CI)		Flexion group versus extension group	
	Flexion group	Extension group	Unadjusted mean differences (95% CI)	Adjusted mean differences (95% CI)*
**Primary outcome**				
**Average pain**				
Baseline	5.99 (5.63–6.38)	5.83 (5.40–6.30)	0.16 (-0.42–0.75)	0.24 (-0.35–0.83)
1 month	4.94 (4.45–5.49)	4.24 (3.53–5.08)	0.71 (-0.22–1.64)	0.65 (-0.29–1.58)
3 months	4.61 (4.03–5.28)	3.05 (2.44–3.82)	1.56 (0.64–2.48)†	1.51 (0.58–2.44)†
6 months	3.75 (3.06–4.59)	3.03 (2.25–4.07)	0.72 (-0.45–1.89)	0.64 (-0.55–1.84)
12 months	3.65 (3.16–4.22)	1.96 (1.46–2.62)	1.70 (0.92–2.48)†	1.65 (0.85–2.44)†
**Secondary outcome: pain subscales**				
**Current pain**				

Baseline	4.77 (4.16–5.47)	4.98 (4.58–5.41)	-0.21 (-0.98–0.57)	-0.25 (-0.99–0.49)
1 month	4.77 (4.16–5.46)	2.81 (2.15–3.67)	1.96 (0.97–2.95)†	1.87 (0.91–2.84)†
3 months	4.23 (3.57–5.01)	2.21 (1.66–2.94)	2.02 (1.06–2.98)†	1.93 (0.97–2.90)†
6 months	3.87 (3.30–4.53)	1.92 (1.30–2.82)	1.95 (0.99–2.91)†	1.89 (0.92–2.85)†
12 months	3.69 (3.11–4.38)	1.61 (0.95–2.71)	2.08 (1.04–3.13)†	2.03 (0.96–3.09)†
**Worst pain**				
Baseline	6.70 (6.29–7.15)	6.11 (5.63–6.64)	0.59 (-0.08–1.26)	0.69 (-0.01–1.39)
1 month	6.00 (5.44–6.62)	5.21 (4.52–6.00)	0.79 (-0.15–1.73)	0.85 (-0.09–1.79)
3 months	5.63 (4.98–6.36)	4.39 (3.66–5.27)	1.24 (0.18–2.29)†	1.21 (0.16–2.26)†
6 months	4.41 (3.67–5.29)	3.75 (2.89–4.85)	0.66 (-0.59–1.92)	0.65 (-0.60–1.89)
12 months	4.4 (3.67–5.29)	3.95 (3.08–5.07)	0.45 (-0.82–1.72)	0.40 (-0.92–1.72)
**Least pain**				
Baseline	2.31 (1.93–2.76)	2.20 (1.64–2.93)	0.11 (-0.65–0.87)	0.01 (-0.71–0.74)

1 month	2.50 (2.02–3.09)	1.62 (1.14–2.3)	0.89 (0.11–1.66)†	0.75 (0.00–1.51)
3 months	2.45 (1.94–3.08)	1.18 (0.85–1.64)	1.26 (0.58–1.95)†	1.14 (0.48–1.81)†
6 months	1.74 (1.36–2.22)	1.11 (0.77–1.59)	0.63 (0.05–1.21)†	0.55 (-0.02–1.12)†
12 months	1.72 (1.37–2.18)	0.75 (0.41–1.37)	0.97 (0.37–1.58)†	0.89 (0.27–1.50)†
**Pain interference**				
Baseline	4.68 (4.03–5.43)	3.56 (2.97–4.28)	1.11 (0.16–2.07)†	1.19 (0.20–2.17)†
1 month	4.27 (3.65–4.99)	3.01 (2.4–3.77)	1.26 (0.31–2.21)†	1.28 (0.31–2.26)†
3 months	3.69 (2.98–4.56)	2.24 (1.73–2.91)	1.45 (0.47–2.42)†	1.46 (0.45–2.47)†
6 months	3.14 (2.45–4.03)	2.55 (1.82–3.57)	0.59 (-0.57–1.75)	0.59 (-0.57–1.76)
12 months	3.39 (2.72–4.22)	1.74 (1.12–2.72)	1.64 (0.57–2.71)†	1.66 (0.55–2.78)†

Generalized linear mixed models comparing between-group difference were used for multiple comparison. Values were presented as least-squares mean (95% CI).

Scores of primary and secondary outcome range from 0 to 10, with higher values indicating more severe pain or interference on daily activity.

CI: confidence interval.

*Adjusted mean between-group difference at the time point was estimated after adjustments for age, sex, and body mass index at baseline.

†*p* for between-group difference <0.05.

Supplementary Table 4. Functional scales at all measure points assessed by LOCF method

	Unadjusted mean (95% CI)		Flexion group versus extension group	
	Flexion group	Extension group	Unadjusted mean differences (95% CI)	Adjusted mean differences (95% CI)*
**ODI**				
Baseline	16.97 (14.18–20.31)	13.41 (11.48–15.68)	3.56 (-0.14–7.25)	3.64 (-0.27–7.55)
1 month	14.89 (12.81–17.32)	13.54 (11.07–16.57)	1.35 (-2.18–4.89)	1.29 (-2.58–5.17)
3 months	14.19 (11.79–17.08)	11.2 (9.19–13.64)	2.99 (-0.44–6.43)	2.95 (-0.39–6.28)
6 months	13.15 (10.38–16.66)	10.9 (7.93–14.97)	2.25 (-2.40–6.91)	2.17 (-2.42–6.76)
12 months	13.16 (10.54–16.44)	10.67 (7.66–14.85)	2.49 (-2.09–7.08)	2.41 (-2.10–6.93)
**EQ-5D**				
Baseline	0.70 (0.66–0.75)	0.75 (0.72–0.79)	-0.05 (-0.11–0.01)	-0.06 (-0.11–0.00)
1 month	0.73 (0.69–0.77)	0.80 (0.77–0.83)	-0.07 (-0.12–-0.02)[†]	-0.07 (-0.12–-0.02)[†]

3 months	0.77 (0.73–0.81)	0.81 (0.78–0.85)	-0.04 (-0.10–0.01)	-0.04 (-0.10–0.01)
6 months	0.79 (0.76–0.83)	0.80 (0.74–0.87)	-0.01 (-0.08–0.06)	-0.01 (-0.08–0.06)
12 months	0.78 (0.75–0.81)	0.80 (0.74–0.86)	-0.02 (-0.09–0.05)	-0.02 (-0.08–0.04)
**PASE**				
Baseline	112.56 (89.62–141.36)	91.82 (78.68–107.15)	20.74 (-8.57–50.04)	20.61 (-6.52–47.75)
1 month	112.86 (91.66–138.96)	105.24 (89.27–124.06)	7.62 (-21.55–36.80)	8.71 (-21.29–38.70)
3 months	119.09 (91.54–154.93)	94.77 (75.51–118.93)	24.32 (-13.69–62.33)	23.39 (-10.51–57.30)
6 months	117.90 (96.41–144.18)	126.49 (96.77–165.34)	-8.59 (-49.95–32.77)	-9.61 (-51.64–32.42)
12 months	118.18 (94.90–147.17)	131.40 (100.90–171.12)	-13.22 (-56.55–30.10)	-13.93 (-55.94–28.07)

Generalized linear mixed models comparing between-group difference were used for multiple comparison. Values were presented as least-squares mean (95% CI).

CI: confidence interval; EQ-5D: EuroQol life-quality index; LOCF: last observation carried forward; ODI: Oswestry disability index; PASE: physical activity scale for the elderly.

*Adjusted mean between-group difference at the time point was estimated after adjustments for age, sex, and body mass index at baseline.

†p for between-group difference <0.05.

Supplementary Table 5. Functional scales at all measure points assessed by mean imputation method

	Unadjusted mean (95% CI)		Flexion group versus extension group	
	Flexion group	Extension group	Unadjusted mean differences (95% CI)	Adjusted mean differences (95% CI)*
**ODI**				
Baseline	18.08 (15.18–21.53)	13.92 (11.96–16.20)	4.16 (0.36–7.96)	4.37 (0.46–8.27)
1 month	15.61 (13.12–18.57)	14.11 (11.61–17.15)	1.50 (-2.37–5.36)	1.49 (-2.63–5.60)
3 months	14.32 (11.66–17.58)	10.87 (8.87–13.33)	3.45 (-0.23–7.13)	3.47 (-0.15–7.09)
6 months	11.74 (9.31–14.79)	11.03 (7.63–15.96)	0.71 (-4.19–5.60)	0.58 (-4.21–5.37)
12 months	11.63 (9.47–14.27)	9.45 (6.92–12.90)	2.18 (-1.61–5.96)	2.23 (-1.81–6.27)
**EQ-5D**				
Baseline	0.70 (0.66–0.75)	0.75 (0.72–0.79)	-0.05 (-0.11–0.00)	-0.06 (-0.11–0.00)
1 month	0.73 (0.69–0.77)	0.80 (0.78–0.83)	-0.07 (-0.12–-0.02)[†]	-0.07 (-0.12–-0.02)[†]

3 months	0.77 (0.73–0.81)	0.82 (0.78–0.86)	-0.05 (-0.11–0.01)	-0.05 (-0.11–0.01)
6 months	0.80 (0.76–0.84)	0.81 (0.73–0.89)	-0.01 (-0.09–0.08)	-0.01 (-0.09–0.08)
12 months	0.79 (0.75–0.82)	0.82 (0.78–0.86)	-0.03 (-0.08–0.02)	-0.03 (-0.09–0.02)
**PASE**				
Baseline	109.02 (86.8–136.94)	94.48 (81.17–109.97)	14.55 (-14.15–43.24)	15.41 (-11.98–42.8)
1 month	108.76 (88.3–133.97)	108.48 (92.41–127.34)	0.29 (-28.29–28.86)	2.45 (-27.33–32.23)
3 months	120.12 (89.61–161.02)	93.94 (72.43–121.83)	26.19 (-16.66–69.03)	24.82 (-13.52–63.17)
6 months	116.05 (94.43–142.62)	133.93 (100.67–178.17)	-17.88 (-62.98–27.21)	-18.66 (-64.94–27.61)
12 months	113.65 (78.41–164.75)	110.57 (72.79–167.97)	3.08 (-59.51–65.67)	-1.74 (-60.73–57.25)

Generalized linear mixed models comparing between-group difference were used for multiple comparison. Values were presented as least-squares mean (95% CI).

CI: confidence interval; EQ-5D: EuroQol life-quality index; LOCF: last observation carried forward; ODI: Oswestry disability index; PASE: physical activity scale for the elderly.

*Adjusted mean between-group difference at the time point was estimated after adjustments for age, sex, and body mass index at baseline.

†*p* for between-group difference <0.05.

Supplementary Table 6. Adherence rate over 1-year follow up

	Flexion group	Extension group	*p* value
1 month	92.59	93.10	0.941
2 months	88.89	89.65	0.926
3 months	87.50	85.19	0.568
4 months	62.50	62.96	0.973
5 months	66.67	61.53	0.468
6 months	63.63	57.69	0.452
12 months	40.00	42.86	0.552

Proportion of adherent participants was estimated. An adherent participant was defined as those who completed their exercises over 30 min for at least 4 times a week.

Supplementary Fig. 1. Exercise interventions based on the lumbar flexion

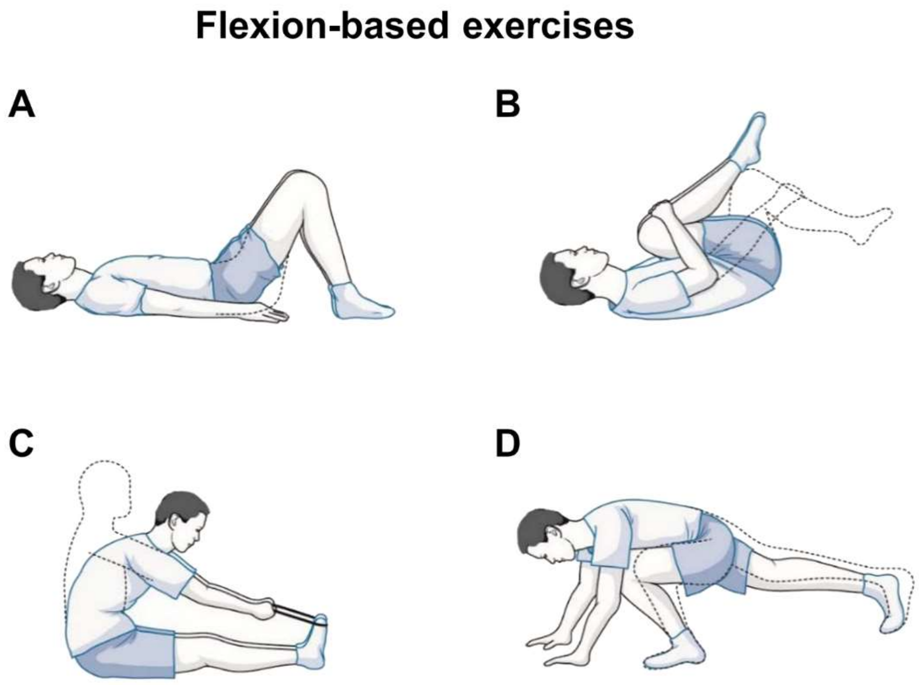

Flexion-based exercises were as follows[1-3].

(A) Pelvic tilt: lie on the back with knees bent and feet flat on the floor. Tighten the abdominal muscles to flatten the back against the floor without pushing down with the legs. The position was held for 10 s (three sets of 10 repetitions).

(B) Knee-to-chest: from a lying position with knees and hips flexed, drawing both knees to the chest while holding both legs. Keep the knees together and the shoulders flat on the floor and hold the position for 10–15 s (three sets of 10 repetitions).

(C) Trunk flexion: seated on the floor with both legs straight, slowly bending forward as far as possible while keeping the legs straight. Slide the hands to the toes until tension is felt in the back and hamstring and hold it for 10 s (three sets of 10 repetitions). If it was difficult to reach the hands to the toes, a towel was wrapped around the feet and pulled to obtain maximum trunk flexion.

(D) Forward bending with hip flexor stretch: forward lunge in a standing position with the feet

shoulder-width apart. Take a big step forward with the left leg and plant the foot out front with the right knee straight and the left knee bent. Bend forward over the left knee to stretch the right hip flexors and hold the position for 10–15 s (three sets of 10 repetitions). Switch legs to stretch the left hip flexors.

Supplementary Fig. 2. Exercise interventions based on the lumbar extension

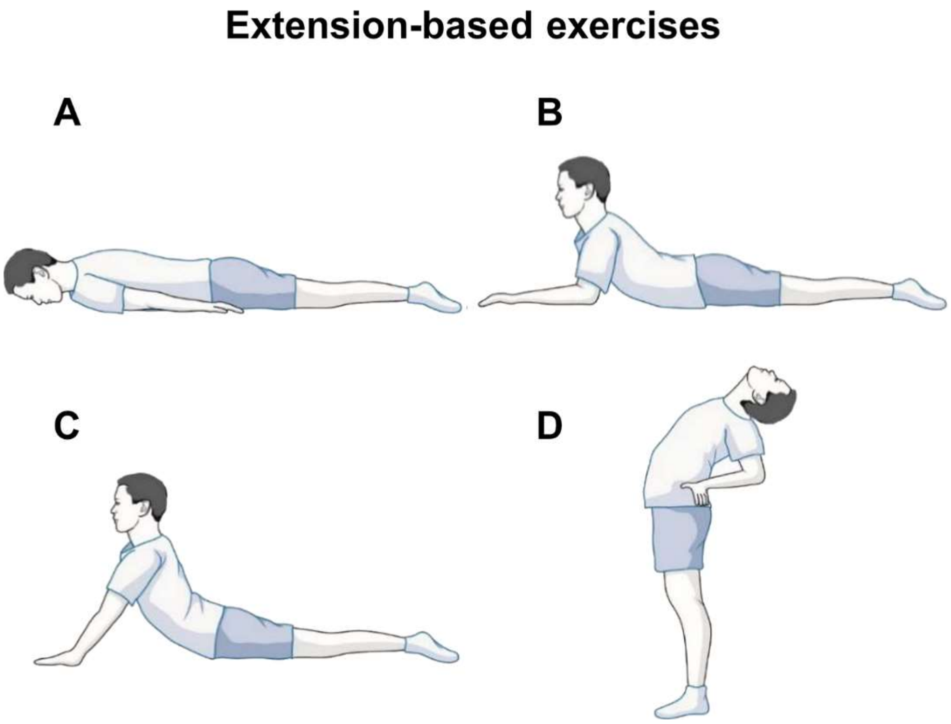

Extension-based exercises were as follows[2-4].

(A) Prone lying flat: in the prone position lying flat on your stomach, put your arms along your sides with the head turned to one side. Maintain this position with the trunk relaxed for 5–10 min.

(B) Prone propped on elbows: lie flat on your stomach and prop yourself on your forearms and elbows with lumbar extension. Remain in this position for 5–10 min.

(C) Prone propped on hands: lie flat on your stomach and prop yourself onto the hands with elbows in full extension, with the trunk fully extended. This position was held for 5–10 s (three sets of 10 repetitions).

(D) Standing lumbar extension: Place both hands at the base of the lower back with feet shoulder-width apart. The trunk was extended backwards as far as possible, with the neck relaxed. The position was held for 5–10 s (three sets of 10 repetitions).

[REFERENCES]

1. Williams PC. Examination and conservative treatment for disk lesions of the lower spine. Clin Orthop 1955;5:28-40.
2. ELNAGGAR IM; NORDIN M; SHEIKHZADEH A; PARNIANPOUR M; KAHANOVITZ N. Effects of spinal flexion and extension exercises on low-back pain and spinal mobility in chronic mechanical low-back pain patients. Spine 1991;16:967-72.
3. Nwuga G; Nwuga V. Relative therapeutic efficacy of the Williams and McKenzie protocols in back pain management. Physiotherapy Practice 2009;1:99-105.
4. McKenzie R; May S. The lumbar spine: mechanical diagnosis & therapy; Spinal Publications, Wellington, 1981.
